# Rates and Factors Associated With Documentation of Diagnostic Codes for Long COVID in the National Veterans Affairs Health Care System

**DOI:** 10.1001/jamanetworkopen.2022.24359

**Published:** 2022-07-29

**Authors:** George N. Ioannou, Aaron Baraff, Alexandra Fox, Troy Shahoumian, Alex Hickok, Ann M. O’Hare, Amy S. B. Bohnert, Edward J. Boyko, Matthew L. Maciejewski, C. Barrett Bowling, Elizabeth Viglianti, Theodore J. Iwashyna, Denise M. Hynes

**Affiliations:** 1 Health Services Research and Development, Center of Innovation, Veterans Affairs Puget Sound Healthcare System, Seattle, Washington; 2 Division of Gastroenterology, Department of Medicine, University of Washington, Seattle; 3 Seattle Epidemiologic Research and Information Center, Veterans Affairs Puget Sound Health Care System, Seattle, Washington; 4 Department of Veterans Affairs, Population Health, Palo Alto Healthcare System, Palo Alto, California; 5 Center of Innovation to Improve Veteran Involvement in Care, VA Portland Healthcare System, Portland, Oregon; 6 Nephrology Section, Veterans Affairs Puget Sound Healthcare System, Seattle, Washington; 7 Division of Nephrology, University of Washington, Seattle; 8 Department of Psychiatry, University of Michigan Medical School, Ann Arbor; 9 General Internal Medicine, Veterans Affairs Puget Sound Healthcare System, Seattle, Washington; 10 Division of General Internal Medicine, University of Washington, Seattle; 11 Center of Innovation to Accelerate Discovery and Practice Transformation, Durham Veterans Affairs Health Care System, Durham, North Carolina; 12 Department of Population Health Sciences, Duke University School of Medicine, Durham, North Carolina; 13 Duke-Margolis Center for Health Policy, Duke University School of Medicine, Durham, North Carolina; 14 Division of General Internal Medicine, Duke University School of Medicine, Durham, North Carolina; 15 Durham Veterans Affairs Geriatric Research Education and Clinical Center, Durham Veterans Affairs Medical Center, Durham, North Carolina; 16 Department of Medicine, Duke University, Durham, North Carolina; 17 Center for Clinical Management Research, VA Ann Arbor Health System, Ann Arbor, Michigan; 18 Division of Pulmonary and Critical Care Medicine, Department of Internal Medicine, University of Michigan, Ann Arbor; 19 Health Management and Policy, School of Social and Behavioral Health Sciences, College of Public Health and Human Sciences, Corvallis, Oregon; 20 Health Data and Informatics Program, Center for Genome Research and Biocomputing, Oregon State University, Corvallis

## Abstract

**Importance:**

Some persons infected with SARS-CoV-2 experience symptoms or impairments many months after acute infection.

**Objectives:**

To determine the rates, clinical setting, and factors associated with documented receipt of COVID-19–related care 3 or more months after acute infection.

**Design, Setting, and Participants:**

This retrospective cohort study used data from the US Department of Veterans Affairs health care system. Participants included persons with a positive SARS-CoV-2 test between February 1, 2020, and April 30, 2021, who were still alive 3 months after infection and did not have evidence of reinfection. Data analysis was performed from February 2020 to December 2021.

**Exposures:**

Positive SARS-CoV-2 test.

**Main Outcomes and Measures:**

Rates and factors associated with documentation of COVID-19–related *International Statistical Classification of Diseases and Related Health Problems, Tenth Revision* codes (U07.1, Z86.16, U09.9, and J12.82) 3 or more months after acute infection (hereafter, long-COVID care), with follow-up extending to December 31, 2021.

**Results:**

Among 198 601 SARS-CoV-2–positive persons included in the study, the mean (SD) age was 60.4 (17.7) years, 176 942 individuals (89.1%) were male, 133 924 (67.4%) were White, 44 733 (22.5%) were Black, and 19 735 (9.9%) were Hispanic. During a mean (SD) follow-up of 13.5 (3.6) months, long-COVID care was documented in a wide variety of clinics, most commonly primary care and general internal medicine (18 634 of 56 310 encounters [33.1%]), pulmonary (7360 of 56 310 encounters [13.1%]), and geriatrics (5454 of 56 310 encounters [9.7%]). Long-COVID care was documented in 26 745 cohort members (13.5%), with great variability across geographical regions (range, 10.8%-18.1%) and medical centers (range, 3.0%-41.0%). Factors significantly associated with documented long-COVID care included older age, Black or American Indian/Alaska Native race, Hispanic ethnicity, geographical region, high Charlson Comorbidity Index score, having documented symptoms at the time of acute infection (adjusted odds ratio [AOR], 1.71; 95% CI, 1.65-1.78) and requiring hospitalization (AOR, 2.60; 95% CI, 2.51-2.69) or mechanical ventilation (AOR, 2.46; 95% CI, 2.26-2.69). Patients who were fully vaccinated at the time of infection were less likely to receive long-COVID care (AOR, 0.78; 95% CI, 0.68-0.90).

**Conclusions and Relevance:**

Long-COVID care was documented in a variety of clinical settings, with great variability across regions and medical centers and was documented more commonly in older persons, those with higher comorbidity burden, those with more severe acute COVID-19 presentation and those who were unvaccinated at the time of infection. These findings provide support and guidance for health care systems to develop systematic approaches to the evaluation and management of patients who may be experiencing long COVID.

## Key Points

**Question:**

What are the rates, clinical settings, and factors associated with documentation of care related to COVID-19 at 3 or more months after acute infection?

**Findings:**

In this cohort study of 198 601 persons with a positive SARS-CoV-2 test, COVID-19 care was documented in 13.5% of individuals 3 or more months after infection during a mean follow-up of 13.5 months and was documented more commonly in older persons, those with higher comorbidity burden, those with more severe acute COVID-19 presentation, and those who were unvaccinated at the time of infection.

**Meaning:**

These findings provide guidance for health care systems to develop systematic approaches to the evaluation and management of patients who may be experiencing long COVID.

## Teaser

This cohort study examines the rates, clinical setting, and factors associated with documented receipt of COVID-19–related care 3 or more months after acute infection among veterans treated in the US Department of Veterans Affairs health care system.

## Introduction

Some patients with acute SARS-CoV-2 infection experience symptoms related to COVID-19 for many months following acute infection. The World Health Organization developed a definition of post–COVID-19 condition (also referred to as long COVID or postacute sequelae of COVID-19) based on certain symptoms or impairments that cannot be explained by an alternative diagnosis being present at least 3 months after the onset of infection.^1^ An analysis^2^ of 456 000 patients attending general practices in England after COVID-19 demonstrated higher general practitioner consultation rates for potential COVID-19 sequelae, most commonly loss of sense of smell or taste, venous thromboembolism, lung fibrosis, breathlessness, joint and muscle pain, anxiety, and kidney impairment. Similar to the protean presentation of acute COVID-19, long COVID may involve multiple organ systems.^3^ As many as 33 postacute sequelae of SARS-CoV-2 infection have been identified,^4^ including pulmonary, cardiovascular, cerebrovascular, thromboembolic, neurocognitive, mental health, metabolic, kidney, and gastrointestinal disorders.^4,5,6,7,8,9,10,11,12,13^

Limited information is available about which patients seek care for potential manifestations of long COVID, the extent to which health care practitioners document care as management of long COVID, or who is providing such care. We examined the rates, clinical setting, and factors associated with receipt of COVID-19–related care 3 or more months after acute infection as evidenced by documentation of COVID-19–specific *International Statistical Classification of Diseases and Related Health Problems, Tenth Revision* (*ICD-10*) codes in the national US Veterans Affairs (VA) health care system.

## Methods

### Data Source and Study Population

The VA is the largest integrated national health care system in the US, providing care at 171 medical centers throughout the country. We used data from the VA’s Corporate Data Warehouse^14^ and the COVID-19 Shared Data Resource, which include analytical variables on all VA enrollees who were tested for SARS-CoV-2, derived from the VA’s comprehensive electronic health record (EHR) system.^15^

We identified all VA enrollees who had documentation in the VA EHR of a positive SARS-CoV-2 RNA polymerase chain reaction test in a respiratory specimen between February 1, 2020, and April 30, 2021 (227 713 enrollees). We excluded 11 907 individuals who died within 3 months of testing positive, and 13 436 who did not have at least 1 primary care encounter in the VA in the 18 months before testing positive. In addition, we excluded 3996 who had a second positive SARS-CoV-2 test 3 or more months after the first so that treatment of reinfections was not incorrectly classified as long-COVID care. The earliest date of a documented positive test was taken as the date of infection.

This cohort study was approved by the VA Puget Sound Institutional Review Board, which waived the requirement to obtain informed consent because this was a retrospective study of EHRs. The study followed the Strengthening the Reporting of Observational Studies in Epidemiology (STROBE) reporting guidelines for cohort studies.

### Outcome Ascertainment

The study’s outcome was defined as documentation in the VA EHR of any of the following 4 COVID-19–related *ICD-10 *codes in 1 or more VA encounters 3 or more months after the date of infection extending to December 31, 2021, henceforth referred to as having documented long-COVID care: U07.1 (“COVID-19”), Z86.16 (“Personal history of COVID-19”), U09.9 (“Post COVID-19 condition, unspecified”), and J12.82 (“Pneumonia due to coronavirus disease 2019”). Although *ICD-10* code U09.9 is specific for post–COVID-19 conditions, it was not introduced until October 1, 2021. All study participants had 1 or more of these 4 *ICD-10 *codes recorded within the first 3 months after infection.

Follow-up for documenting long-COVID care extended from 8 months (ie, if testing positive on April 30, 2021) to 23 months (ie, if testing positive on February 1, 2020). A secondary analysis was performed with follow-up limited to 8 months (240 days) from the date of infection such that all participants would have the same duration of follow-up.

### Baseline Characteristics

We ascertained sociodemographic (including race and ethnicity), geographical, and clinical characteristics, based on a 2-year lookback window, that were potentially associated with long-COVID care documentation (Table 1). The *ICD-10* codes used to define each comorbid condition were provided by the VA Centralized Interactive Phenomics Resource.^16^

**Table 1.** Baseline Characteristics of Veterans Affairs Health System Enrollees Who Tested Positive for SARS-CoV-2 Infection From February 2020 to April 2021, According to Whether They Had COVID-19 *ICD-10* Codes Documented 3 or More Months After First Testing Positive for SARS-CoV-2 Infection, With Follow-up Extending to December 31, 2021

Baseline characteristics	Patients, No. (%)		
	COVID-19 *ICD-10* codes documented ≥3 mo after testing positive for SARS-CoV-2 infection		Total (N = 198 601)
	No (n = 171 856)	Yes (n = 26 745)	
Sociodemographic characteristics			
Age, y			
18-49	47 015 (27.4)	5208 (19.5)	52 223 (26.3)
50-69	31 598 (18.4)	4887 (18.3)	36 485 (18.4)
60-64	17 851 (10.4)	2968 (11.1)	20 819 (10.5)
65-69	17 497 (10.2)	3174 (11.9)	20 671 (10.4)
70-74	29 712 (17.3)	5337 (20.0)	35 049 (17.6)
75-79	13 926 (8.1)	2587 (9.7)	16 513 (8.3)
80-84	6503 (3.8)	1256 (4.7)	7759 (3.9)
85-89	4833 (2.8)	842 (3.1)	5675 (2.9)
≥90	2910 (1.7)	485 (1.8)	3395 (1.7)
Sex			
Male	152 895 (89.0)	24 047 (89.9)	176 942 (89.1)
Female	18 961 (11.0)	2698 (10.1)	21 659 (10.9)
Race			
African American or Black	38 165 (22.2)	6568 (24.6)	44 733 (22.5)
American Indian or Alaska Native	1565 (0.9)	263 (1.0)	1828 (0.9)
Asian	1721 (1.0)	243 (0.9)	1964 (1.0)
Native Hawaiian or Pacific Islander	1625 (0.9)	256 (1.0)	1881 (0.9)
White	116 454 (67.8)	17 470 (65.3)	133 924 (67.4)
Declined or missing	12 326 (7.2)	1945 (7.3)	14 271 (7.2)
Ethnicity			
Not Hispanic or Latino	148 845 (86.6)	22 877 (85.5)	171 722 (86.5)
Hispanic or Latino	16 790 (9.8)	2945 (11.0)	19 735 (9.9)
Declined or missing	6221 (3.6)	923 (3.5)	7144 (3.6)
Rural vs urban residence			
Rural	26 917 (15.7)	3409 (12.7)	30 326 (15.3)
Urban	119 847 (69.7)	18 435 (68.9)	138 282 (69.6)
Unknown	25 092 (14.6)	4901 (18.3)	29 993 (15.1)
VA Integrated Service Network			
1	5886 (3.4)	864 (3.2)	6750 (3.4)
2	7422 (4.3)	1382 (5.2)	8804 (4.4)
4	7586 (4.4)	1154 (4.3)	8740 (4.4)
5	4543 (2.6)	895 (3.3)	5438 (2.7)
6	10 856 (6.3)	1310 (4.9)	12 166 (6.1)
7	14 956 (8.7)	1953 (7.3)	16 909 (8.5)
8	13 226 (7.7)	2426 (9.1)	15 652 (7.9)
9	8108 (4.7)	1075 (4.0)	9183 (4.6)
10	13 374 (7.8)	2157 (8.1)	15 531 (7.8)
12	8296 (4.8)	1443 (5.4)	9739 (4.9)
15	8335 (4.8)	1180 (4.4)	9515 (4.8)
16	11 823 (6.9)	1579 (5.9)	13 402 (6.7)
17	12 175 (7.1)	2688 (10.1)	14 863 (7.5)
19	8213 (4.8)	1333 (5.0)	9546 (4.8)
20	4425 (2.6)	648 (2.4)	5073 (2.6)
21	7304 (4.3)	1212 (4.5)	8516 (4.3)
22	15 000 (8.7)	2231 (8.3)	17 231 (8.7)
23	10 325 (6.0)	1215 (4.5)	11 540 (5.8)
Time period of infection			
Before June 1, 2020 (first wave)	9440 (5.5)	2184 (8.2)	11 624 (5.9)
June 1 to October 31, 2020 (second wave)	40 618 (23.6)	7188 (26.9)	47 806 (24.1)
November 1, 2020, to April 30, 2021 (third wave/Alpha variant)	121 798 (70.9)	17 373 (65.0)	139 171 (70.1)
Comorbid conditions			
Charlson Comorbidity Index score			
0	70 635 (41.1)	7703 (28.8)	78 338 (39.4)
1	36 204 (21.1)	5399 (20.2)	41 603 (20.9)
2	25 588 (14.9)	4196 (15.7)	29 784 (15.0)
3	14 044 (8.2)	2829 (10.6)	16 873 (8.5)
4	9581 (5.6)	2149 (8.0)	11 730 (5.9)
5-6	9910 (5.8)	2548 (9.5)	12 458 (6.3)
7-8	4101 (2.4)	1249 (4.7)	5350 (2.7)
≥9	1793 (1.0)	672 (2.5)	2465 (1.2)
Diabetes	57 147 (33.3)	10 826 (40.5)	67 973 (34.2)
Chronic obstructive pulmonary disease	23 863 (13.9)	5895 (22.0)	29 758 (15.0)
Asthma	11 890 (6.9)	2542 (9.5)	14 432 (7.3)
Congestive heart failure	10 716 (6.2)	2967 (11.1)	13 683 (6.9)
Myocardial infarction	3268 (1.9)	870 (3.3)	4138 (2.1)
Cerebrovascular disease	2966 (1.7)	764 (2.9)	3730 (1.9)
Chronic kidney disease	20 946 (12.2)	4850 (18.1)	25 796 (13.0)
Peripheral arterial disease	16 014 (9.3)	3928 (14.7)	19 942 (10.0)
Venous thromboembolism	3843 (2.2)	1021 (3.8)	4864 (2.4)
Obstructive sleep apnea	55 101 (32.1)	10 199 (38.1)	65 300 (32.9)
Obesity hypoventilation syndrome	696 (0.4)	217 (0.8)	913 (0.5)
Depression	58 117 (33.8)	9878 (36.9)	67 995 (34.2)
Posttraumatic stress disorder	43 089 (25.1)	7067 (26.4)	50 156 (25.3)
Bipolar-schizophrenia	9020 (5.2)	1657 (6.2)	10 677 (5.4)
Medications			
Opioids	8618 (5.0)	2166 (8.1)	10 784 (5.4)
Antidepressants	55 382 (32.2)	9477 (35.4)	64 859 (32.7)
Statins	85 447 (49.7)	15 471 (57.8)	100 918 (50.8)
Angiotensin-converting enzyme inhibitors	49 698 (28.9)	8899 (33.3)	58 597 (29.5)
Angiotensin receptor blockers	23 757 (13.8)	4603 (17.2)	28 360 (14.3)
Calcium channel blockers	61 839 (36.0)	12 004 (44.9)	73 843 (37.2)
Severity of acute SARS-CoV-2 infection			
Hospitalization within 30 d of infection	15 145 (8.8)	6297 (23.5)	21 442 (10.8)
Mechanical ventilation for acute infection	1577 (0.9)	794 (3.0)	2371 (1.2)
Symptoms at presentation with acute infection, No.			
0	89 534 (52.1)	10 386 (38.8)	99 920 (50.3)
1-2	35 741 (20.8)	6675 (25.0)	42 416 (21.4)
3-4	24 304 (14.1)	5203 (19.5)	29 507 (14.9)
≥5	22 276 (13.0)	4481 (16.8)	26 757 (13.5)
Vaccine doses received at the time of infection, No.^a^			
0	51 882 (87.6)	6811 (86.8)	58 693 (87.5)
1	5138 (8.7)	772 (9.8)	5910 (8.8)
2	2184 (3.7)	263 (3.4)	2447 (3.6)
Healthcare utilization			
Primary care visits in prior 2 y, No.			
0-5	83 639 (48.7)	10 444 (39.1)	94 083 (47.4)
6-11	48 238 (28.1)	7761 (29.0)	55 999 (28.2)
≥12	38 806 (22.6)	8354 (31.2)	47 160 (23.7)
Mental health visits in prior 2 y, No.			
0	95 998 (55.9)	14 059 (52.6)	110 057 (55.4)
1-6	34 186 (19.9)	5369 (20.1)	39 555 (19.9)
7-19	23 597 (13.7)	3864 (14.4)	27 461 (13.8)
≥20	16 902 (9.8)	3267 (12.2)	20 169 (10.2)
Specialty care visits in prior 2 y, No.			
0	3424 (2.0)	322 (1.2)	3746 (1.9)
1-9	83 336 (48.5)	9646 (36.1)	92 982 (46.8)
10-18	45 155 (26.3)	7221 (27.0)	52 376 (26.4)
≥19	38 768 (22.6)	9370 (35.0)	48 138 (24.2)

Abbreviation: *ICD-10*, *International Statistical Classification of Diseases and Related Health Problems, Tenth Revision*.

^a^ For COVID-19 vaccination, we limited analyses to persons infected after January 1, 2021, when vaccines became widely available. We excluded from vaccination analyses a very small proportion of vaccine recipients (0.6%) who received the Janssen (JNJ-78436735) vaccine.

We determined whether 1 or 2 mRNA COVID-19 vaccine doses (ie, mRNA-1273 by Moderna or BNT162b2 by Pfizer-BioNTech) were administered before the date of infection. We identified both vaccinations performed within VA through pharmacy records (84.9% of cases) as well as vaccinations performed outside the VA (15.1% of cases) confirmed by documentation of type and date of vaccination in VA records. For each patient, we ascertained the number of primary care, mental health, and specialty outpatient encounters during the 2-year period before infection.

### Characteristics Related to the Severity of the Acute SARS-CoV-2 Infection

We ascertained 15 prespecified symptoms present at the time of testing positive or within the preceding 30 days, extracted from the EHR by the Veterans Affairs Informatics and Computing Infrastructure COVID-19 Shared Data Resource natural language processing team using a combination of all relevant outpatient and inpatient clinical notes, COVID-19 symptom screening questionnaires, vital signs, and relevant *ICD-10* codes for symptoms. These symptoms could be related to COVID-19 but could also potentially be related to preexisting conditions. We identified whether SARS-CoV-2–infected persons were hospitalized in the VA health care system within 30 days after testing positive and whether those hospitalized underwent mechanical ventilation.

### Statistical Analysis

We evaluated whether patient characteristics were associated with the outcome of long-COVID care using multivariable logistic regression with adjustment for age, sex, self-reported race, self-reported ethnicity, urban vs rural residence (based on zip codes, using data from the VA Office of Rural Health,^17^ which uses the Secondary Rural-Urban Commuting Area for defining rurality), Charlson Comorbidity Index (CCI) score, VA Integrated Service Network (VISN, or the VA’s administrative regions^18^), time period of infection (categorized by pandemic waves), and number of primary care, mental health, and specialty care encounters in the 2 years before infection; all models are outlined in eTable 1 in the Supplement. Results are presented as crude and adjusted odds ratios (ORs), with a 95% CI. By adjusting for the number of encounters before infection we hoped to account for the propensity to have encounters after infection during which COVID-19–specific codes would be more likely to be documented.

When we evaluated individual comorbidities, we did not simultaneously adjust for CCI score because it would result in overadjustment as the CCI captures multiple comorbid conditions. Analyses of COVID-19 vaccination status were limited to persons infected after January 1, 2021 (67 050 individuals), when vaccines became widely available, and were adjusted for time of infection in monthly time periods, to account for rapid changes in vaccination status.

When investigating time period of infection, we limited the outcome to COVID-19 *ICD-10* codes documented from 90 days to 240 days after infection such that all time periods had equal duration of follow-up. Data analysis was performed from February 2020 to December 2021. Data were analyzed with Stata statistical software version 16 (StataCorp).

## Results

### Characteristics of the Study Population

Our cohort of 198 601 individuals had a mean (SD) age 60.4 (17.7) years (79 992 individuals [45.0%] were aged ≥65 years), 176 942 individuals (89.1%) were men, 133 924 (67.4%) were White, 44 733 (22.5%) were Black, and 19 735 (9.9%) were Hispanic. There was a high prevalence of comorbid conditions (Table 1).

During a mean (SD) follow-up of 13.5 (3.6) months, long-COVID care was documented in 26 745 individuals (13.5%) overall, including 29.3% (6297 of 21 442 individuals) of those hospitalized within 30 days for acute COVID-19 and 11.5% (20 448 of 177 159 individuals) of those not hospitalized. Compared with patients without documented long-COVID care, those with documented long-COVID care were older, had higher prevalence of multiple comorbid conditions (chronic obstructive pulmonary disease [COPD], congestive heart failure, chronic kidney disease, and diabetes), higher CCI score, higher hospitalization and ventilation rates, and more symptoms at the time of the acute SARS-CoV-2 infection (Table 1).

### Distribution of Diagnostic Codes and Clinics at Which the Long-COVID Codes Were Documented

Among the 26 745 patients with documented long-COVID care in 56 310 encounters, the majority of COVID-19–related *ICD-10 *codes were U07.1 (29 327 individuals [52.48%]) and Z86.16 (24 217 individuals [43.34%]) with only a very small proportion of U09.9 (2212 individuals [3.96%]) and J12.82 (713 individuals [1.28%]) (Table 2). Most patients had long-COVID care documented only once (16 343 of 26 745 individuals [61.1%]) and 2 to 5 times (8630 of 26 745 individuals [32.2%]) (Table 2).

**Table 2.** Characteristics of Encounters That Documented the *ICD-10* Codes for COVID-19 3 or More Months After Testing Positive for Acute SARS-CoV-2 Infection

Characteristic	Encounters, No./patients, No. (%)
Times an *ICD-10* code for COVID-19 was recorded ≥3 mo after the index date, No.	
1	16 343/26 745 (61.1)
2-5	8630/26 745 (32.3)
6-10	1227/26 745 (4.6)
11-20	432/26 745 (1.6)
>20	113/26 745 (0.4)
Encounters with an *ICD-10* code for COVID-19 over time since infection, No. (%)	
91-120 d since infection	9960 (17.7)
121-150 d since infection	6993 (12.4)
151-180 d since infection	5698 (10.1)
181-210 d since infection	5543 (9.8)
211-240 d since infection	4713 (8.4)
241-270 d since infection	4505 (8.0)
>270 d since infection	18 898 (33.6)
Distribution of different *ICD-10* codes for COVID-19 recorded ≥3 mo after the index date, No. (%)	
U07.1	29 327 (52.48)
Z86.16	24 217 (43.34)
U09.9	2212 (3.96)
J12.82	713 (1.28)
Distribution of clinics that recorded different* ICD-10* codes for COVID-19 ≥3 mo after the index date	
Primary care and general internal medicine	18 634/56 310 (33.1)
Pulmonary and respiratory therapy	7360/56 310 (13.1)
Geriatrics	5454/56 310 (9.7)
Physical therapy	1821/56 310 (3.2)
Mental health	1944/56 310 (3.5)
Occupational therapy	849/56 310 (1.5)
Infectious diseases	968/56 310 (1.7)
Cardiology	1269/56 310 (2.2)
Rehabilitation medicine	1462/56 310 (2.6)
Nephrology	507/56 310 (0.9)
Neurology	472/56 310 (0.8)

Abbreviation: *ICD-10, International Statistical Classification of Diseases and Related Health Problems, Tenth Revision.*

The most common outpatient clinics (including telehealth clinics) at which long-COVID codes were documented were primary care and general internal medicine (18 634 of 56 310 encounters [33.1%]), pulmonary and respiratory therapy (7360 of 56 310 encounters [13.1%]), geriatrics (5454 of 56 310 encounters [9.7%]), physical therapy (1821 of 56 310 encounters [3.2%]), and mental health (1944 of 56 310 encounters [3.5%]), with much smaller representation in occupational therapy (849 of 56 310 encounters [1.5%]), infectious diseases (968 of 56 310 encounters [1.7%]), cardiology (1269 of 56 310 encounters [2.2%]), rehabilitation medicine (1462 of 56 310 encounters [2.6%]), nephrology (507 of 56 310 encounters [0.9%]), and neurology (472 of 56 310 encounters [0.8%]) (Table 2). There was a gradual decline in the number of encounters with documented long-COVID codes each month, from 9960 at 91 to 120 days after infection to 4505 at 241 to 270 days after infection (Table 2).

### Associations Between Baseline Characteristics and Long-COVID Care

Compared with persons aged 18 to 49 years, older age groups were progressively more likely to have documentation of long-COVID care up to age group 80 to 84 years (adjusted OR [AOR], 1.38; 95% CI, 1.28-1.48), with some decline in older age groups (Table 3 and eFigure in the Supplement). Compared with White patients, Black (AOR, 1.10; 95% CI, 1.08-1.21), Asian (AOR, 1.12; 95% CI, 0.98-1.29), and American Indian/Alaska Native (AOR, 1.18; 95% CI, 1.03-1.35) patients were significantly more likely to have documentation of long-COVID care. Long-COVID care was more likely to be documented in Hispanic (vs non-Hispanic) patients (AOR, 1.15; 95% CI, 1.10-1.21) and those with urban (vs rural) residence (AOR, 1.14, 95% CO 1.10-1.19).

**Table 3.** Associations Between Baseline Characteristics and the Documentation of COVID-19 *ICD-10* Codes 3 or More Months After Testing Positive for SARS-CoV-2 Infection Among Veterans Affairs Health Care System Enrollees Who Tested Positive for SARS-CoV-2 Infection From February 2020 to April 2021 With Follow-up Extending to December 31, 2021

Characteristics	COVID-19 *ICD-10* codes documented ≥3 mo after infection, patients, No. (%) (N = 198 601)		OR (95% CI)	
	No (n = 171 856)	Yes (n = 26 745)	Crude	Adjusted^a^
Sociodemographic characteristics				
Age, y				
18-49	47 015 (90.0)	5208 (10.0)	1 [Reference]	1 [Reference]
50-69	31 598 (86.6)	4887 (13.4)	1.40 (1.34-1.46)	1.25 (1.19-1.30)
60-64	17 851 (85.7)	2968 (14.3)	1.50 (1.43-1.58)	1.22 (1.16-1.28)
65-69	17 497 (84.6)	3174 (15.4)	1.64 (1.56-1.72)	1.28 (1.21-1.35)
70-74	29 712 (84.8)	5337 (15.2)	1.62 (1.56-1.69)	1.28 (1.22-1.34)
75-79	13 926 (84.3)	2587 (15.7)	1.68 (1.59-1.76)	1.32 (1.24-1.39)
80-84	6503 (83.8)	1256 (16.2)	1.74 (1.63-1.86)	1.38 (1.28-1.48)
85-89	4833 (85.2)	842 (14.8)	1.57 (1.45-1.70)	1.26 (1.15-1.37)
≥90	2910 (85.7)	485 (14.3)	1.50 (1.36-1.66)	1.21 (1.09-1.34)
Sex				
Male	152 895 (86.4)	24 047 (13.6)	1 [Reference]	1 [Reference]
Female	18 961 (87.5)	2698 (12.5)	0.90 (0.87-0.94)	1.03 (0.99-1.08)
Race				
American Indian or Alaska Native	1565 (85.6)	263 (14.4)	1.12 (0.98-1.28)	1.18 (1.03-1.35)
African American or Black	38 165 (85.3)	6568 (14.7)	1.15 (1.11-1.18)	1.11 (1.07-1.14)
Asian	1721 (87.6)	243 (12.4)	0.94 (0.82-1.08)	1.12 (0.98-1.29)
Native Hawaiian or Pacific Islander	1625 (86.4)	256 (13.6)	1.05 (0.92-1.20)	1.03 (0.90-1.17)
White	116 454 (87.0)	17 470 (13.0)	1 [Reference]	1 [Reference]
Declined or missing	12 326 (86.4)	1945 (13.6)	1.05 (1.00-1.11)	1.07 (1.01-1.13)
Ethnicity				
Not Hispanic or Latino	148 845 (86.7)	22 877 (13.3)	1 [Reference]	1 [Reference]
Hispanic or Latino	16 790 (85.1)	2945 (14.9)	1.14 (1.09-1.19)	1.15 (1.10-1.21)
Declined or missing	6221 (87.1)	923 (12.9)	0.97 (0.90-1.04)	1.00 (0.92-1.08)
Rural vs urban residence				
Rural	26 917 (88.8)	3409 (11.2)	1 [Reference]	1 [Reference]
Urban	119 847 (86.7)	18 435 (13.3)	1.21 (1.17-1.26)	1.14 (1.10-1.19)
Unknown	25 092 (83.7)	4901 (16.3)	1.54 (1.47-1.62)	1.41 (1.35-1.48)
VISN				
8^b^	13 226 (84.5)	2426 (15.5)	1 [Reference]	1 [Reference]
6	10 856 (89.2)	1310 (10.8)	0.66 (0.61-0.71)	0.69 (0.64-0.74)
23^b^	10 325 (89.5)	1215 (10.5)	0.64 (0.60-0.69)	0.73 (0.67-0.78)
9	8108 (88.3)	1075 (11.7)	0.72 (0.67-0.78)	0.74 (0.68-0.80)
7	14 956 (88.4)	1953 (11.6)	0.71 (0.67-0.76)	0.75 (0.70-0.80)
16^b^	11 823 (88.2)	1579 (11.8)	0.73 (0.68-0.78)	0.76 (0.71-0.82)
1	5886 (87.2)	864 (12.8)	0.80 (0.74-0.87)	0.83 (0.77-0.91)
15	8335 (87.6)	1180 (12.4)	0.77 (0.72-0.83)	0.84 (0.78-0.91)
22^b^	15 000 (87.1)	2231 (12.9)	0.81 (0.76-0.86)	0.87 (0.82-0.93)
20^b^	4425 (87.2)	648 (12.8)	0.80 (0.73-0.88)	0.90 (0.82-0.99)
4	7586 (86.8)	1154 (13.2)	0.83 (0.77-0.89)	0.90 (0.83-0.97)
10	13 374 (86.1)	2157 (13.9)	0.88 (0.83-0.94)	0.91 (0.85-0.97)
12^b^	8296 (85.2)	1443 (14.8)	0.95 (0.88-1.02)	0.96 (0.89-1.03)
2	7422 (84.3)	1382 (15.7)	1.02 (0.94-1.09)	0.97 (0.90-1.05)
21^b^	7304 (85.8)	1212 (14.2)	0.90 (0.84-0.97)	0.98 (0.91-1.06)
19^b^	8213 (86.0)	1333 (14.0)	0.88 (0.82-0.95)	1.01 (0.94-1.09)
5^b^	4543 (83.5)	895 (16.5)	1.07 (0.99-1.17)	1.10 (1.01-1.20)
17^b^	12 175 (81.9)	2688 (18.1)	1.20 (1.13-1.28)	1.31 (1.24-1.40)
Time period of infection^c^				
Before June 1, 2020 (first wave)	10 867 (93.5)	757 (6.5)	1 [Reference]	1 [Reference]
June 1 to October 31, 2020 (second wave)	43 625 (91.3)	4181 (8.7)	1.38 (1.27-1.49)	1.52 (1.40-1.65)
November 1, 2020, to April 30, 2021 (third wave/Alpha variant)	126 378 (90.8)	12 793 (9.2)	1.45 (1.35-1.57)	1.65 (1.52-1.78)
Comorbid conditions				
CCI score				
0	70 635 (90.2)	7703 (9.8)	1 [Reference]	1 [Reference]
1	36 204 (87.0)	5399 (13.0)	1.37 (1.32-1.42)	1.22 (1.18-1.27)
2	25 588 (85.9)	4196 (14.1)	1.50 (1.44-1.57)	1.25 (1.20-1.31)
3	14 044 (83.2)	2829 (16.8)	1.85 (1.76-1.94)	1.44 (1.37-1.52)
4	9581 (81.7)	2149 (18.3)	2.06 (1.95-2.17)	1.54 (1.46-1.64)
5-6	9910 (79.5)	2548 (20.5)	2.36 (2.24-2.48)	1.68 (1.59-1.78)
7-8	4101 (76.7)	1249 (23.3)	2.79 (2.61-2.99)	1.87 (1.73-2.01)
≥9	1793 (72.7)	672 (27.3)	3.44 (3.14-3.77)	2.19 (1.98-2.41)
Body mass index^d^				
<18.5	1358 (83.1)	276 (16.9)	1.20 (1.05-1.38)	1.01 (0.89-1.16)
18.5-25	24 022 (85.6)	4057 (14.4)	1 [Reference]	1 [Reference]
>25-30	55 535 (86.9)	8350 (13.1)	0.89 (0.85-0.93)	0.96 (0.92-1.00)
>30-35	50 392 (87.0)	7499 (13.0)	0.88 (0.85-0.92)	0.96 (0.92-1.00)
>35-40	25 216 (86.3)	4009 (13.7)	0.94 (0.90-0.99)	1.01 (0.96-1.06)
>40	14 228 (85.1)	2493 (14.9)	1.04 (0.98-1.10)	1.09 (1.03-1.15)
Diabetes				
No	114 708 (87.8)	15 919 (12.2)	1 [Reference]	1 [Reference]
Yes	57 147 (84.1)	10 826 (15.9)	1.37 (1.33-1.40)	1.07 (1.04-1.11)
Chronic obstructive pulmonary disease				
No	147 992 (87.7)	20 850 (12.3)	1 [Reference]	1 [Reference]
Yes	23 863 (80.2)	5895 (19.8)	1.75 (1.70-1.81)	1.42 (1.38-1.47)
Asthma				
No	159 965 (86.9)	24 203 (13.1)	1 [Reference]	1 [Reference]
Yes	11 890 (82.4)	2542 (17.6)	1.41 (1.35-1.48)	1.32 (1.26-1.38)
Congestive heart failure				
No	161 139 (87.1)	23 778 (12.9)	1 [Reference]	1 [Reference]
Yes	10 716 (78.3)	2967 (21.7)	1.88 (1.80-1.96)	1.34 (1.28-1.41)
Myocardial infarction				
No	168 587 (86.7)	25 875 (13.3)	1 [Reference]	1 [Reference]
Yes	3268 (79.0)	870 (21.0)	1.73 (1.61-1.87)	1.28 (1.18-1.38)
Cerebrovascular disease				
No	168 889 (86.7)	25 981 (13.3)	1 [Reference]	1 [Reference]
Yes	2966 (79.5)	764 (20.5)	1.67 (1.54-1.81)	1.24 (1.14-1.35)
Chronic kidney disease				
No	150 909 (87.3)	21 895 (12.7)	1 [Reference]	1 [Reference]
Yes	20 946 (81.2)	4850 (18.8)	1.60 (1.54-1.65)	1.22 (1.18-1.27)
Peripheral arterial disease				
No	155 841 (87.2)	22 817 (12.8)	1 [Reference]	1 [Reference]
Yes	16 014 (80.3)	3928 (19.7)	1.68 (1.61-1.74)	1.23 (1.18-1.28)
Venous thromboembolism				
No	168 012 (86.7)	25 724 (13.3)	1 [Reference]	1 [Reference]
Yes	3843 (79.0)	1021 (21.0)	1.74 (1.62-1.86)	1.30 (1.21-1.40)
Obstructive sleep apnea				
No	116 754 (87.6)	16 546 (12.4)	1 [Reference]	1 [Reference]
Yes	55 101 (84.4)	10 199 (15.6)	1.31 (1.27-1.34)	1.16 (1.13-1.19)
Obesity hypoventilation syndrome				
No	171 159 (86.6)	26 528 (13.4)	1 [Reference]	1 [Reference]
Yes	696 (76.2)	217 (23.8)	2.01 (1.73-2.34)	1.48 (1.27-1.73)
Medications				
Opioids				
No	163 238 (86.9)	24 579 (13.1)	1 [Reference]	1 [Reference]
Yes	8618 (79.9)	2166 (20.1)	1.67 (1.59-1.75)	1.24 (1.17-1.30)
Antidepressants				
No	116 474 (87.1)	17 268 (12.9)	1 [Reference]	1 [Reference]
Yes	55 382 (85.4)	9477 (14.6)	1.15 (1.12-1.19)	1.02 (0.99-1.05)
Statins				
No	86 409 (88.5)	11 274 (11.5)	1 [Reference]	1 [Reference]
Yes	85 447 (84.7)	15 471 (15.3)	1.39 (1.35-1.42)	1.00 (0.97-1.03)
Angiotensin-converting enzyme inhibitors				
No	122 158 (87.3)	17 846 (12.7)	1 [Reference]	1 [Reference]
Yes	49 698 (84.8)	8899 (15.2)	1.23 (1.19-1.26)	0.99 (0.96-1.02)
Angiotensin receptor blockers				
No	148 099 (87.0)	22 142 (13.0)	1 [Reference]	1 [Reference]
Yes	23 757 (83.8)	4603 (16.2)	1.30 (1.25-1.34)	1.03 (0.99-1.06)
Calcium channel blockers				
No	110 017 (88.2)	14 741 (11.8)	1 [Reference]	1 [Reference]
Yes	61 839 (83.7)	12 004 (16.3)	1.45 (1.41-1.49)	1.24 (1.20-1.27)
Healthcare utilization				
Primary care visits in prior 2 y, No.				
0-5	83 639 (88.9)	10 444 (11.1)	1 [Reference]	1 [Reference]
6-11	48 238 (86.1)	7761 (13.9)	1.29 (1.25-1.33)	0.99 (0.96-1.03)
≥12	38 806 (82.3)	8354 (17.7)	1.72 (1.67-1.78)	0.95 (0.91-1.00)
Mental health visits in prior 2 y, No.				
0	95 998 (87.2)	14 059 (12.8)	1 [Reference]	1 [Reference]
1-6	34 186 (86.4)	5369 (13.6)	1.07 (1.04-1.11)	1.02 (0.98-1.05)
7-19	23 597 (85.9)	3864 (14.1)	1.12 (1.08-1.16)	1.05 (1.01-1.09)
≥20	16 902 (83.8)	3267 (16.2)	1.32 (1.27-1.38)	1.16 (1.11-1.21)
Specialty care visits in prior 2 y, No.				
0	3424 (91.4)	322 (8.6)	1 [Reference]	1 [Reference]
1-9	83 336 (89.6)	9646 (10.4)	1.23 (1.10-1.38)	1.15 (1.01-1.31)
10-18	45 155 (86.2)	7221 (13.8)	1.70 (1.51-1.91)	1.44 (1.26-1.65)
≥19	38 768 (80.5)	9370 (19.5)	2.57 (2.29-2.89)	1.90 (1.65-2.18)

Abbreviations: CCI, Charlson Comorbidity Index; *ICD-10, International Statistical Classification of Diseases and Related Health Problems, Tenth Revision*; OR, odds ratio; VISN, VA Integrated Service Network.

^a^ Adjusted by multivariable logistic regression for age (using the categories shown), sex, race, ethnicity, urban vs rural residence, CCI, VISN, time period of infection (categorized according to the waves of the pandemic as shown), and number of primary care, mental health and specialty care encounters in the 2 years before infection. When we evaluated the associations of any of the individual comorbidities (eg, chronic obstructive pulmonary disease, congestive heart failure, chronic kidney disease, diabetes, depression, posttraumatic stress disorder, bipolar-schizoaffective disorder, cancer, hypertension, obesity, cerebrovascular disease, smoking, and others), we did not simultaneously adjust for the CCI score because it would result in overadjustment.

^b^ Denotes VISNs that have facilities with established dedicated clinics for the follow-up of patients with long COVID.

^c^ When looking at time period of infection, we limited the outcome to COVID-19 *ICD-10* codes documented from 3 to 8 months after infection such that all time periods had equal length of follow-up.

^d^ Body mass index is calculated as weight in kilograms divided by height in meters squared.

There was substantial variability between VISNs in documentation of long-COVID care, the lowest being VISN 6 (North Carolina and Virginia, 10.8%) and the highest being VISN 17 (Texas, 18.1%). There was even greater variability by facility (medical center), ranging from 3% to 41%, with 16 VA facilities that have established dedicated clinics for long-COVID follow-up having higher rates (Figure). Compared with persons infected during the first wave of the pandemic (ie, before June 1, 2020), those infected between June and October 2020 (AOR, 1.52; 95% CI, 1.40-1.65) or between November 2020 and April 2021 (AOR, 1.65; 95% CI, 1.52-1.78) were more likely to have documented long-COVID care from 3 to 8 months after infection.

**Figure.**
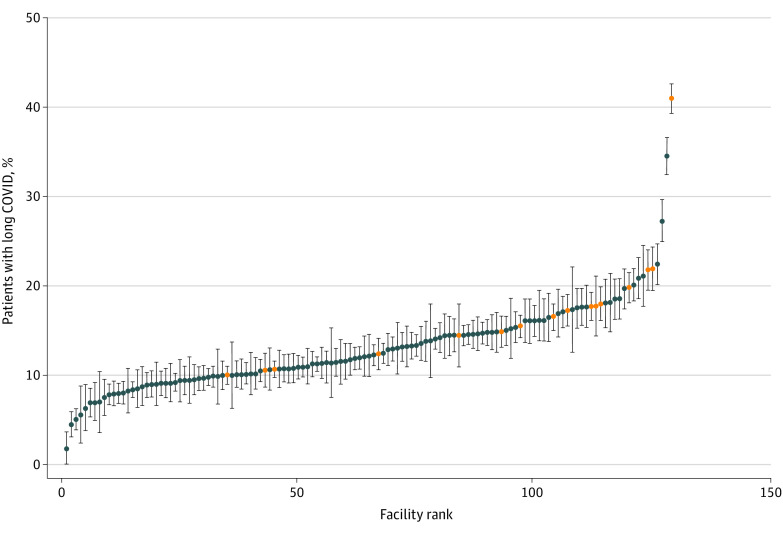
Proportion of SARS-CoV-2–Positive Patients Who Have Documentation of COVID-19 *International Classification of Diseases, Tenth Revision*, Codes 3 or More Months After Testing Positive by Facility (Medical Center) Facilities highlighted in orange are the 16 Veterans Affairs facilities that have established dedicated clinics for the follow-up of patients with long COVID. Circles denote means, and error bars denote 95% CIs.

The CCI score was one of the variables most associated with documentation of long-COVID care, with a linear association seen between CCI score and long-COVID care (Table 3 and eFigure in the Supplement). Comorbid conditions associated with long-COVID care included COPD, asthma, congestive heart failure, prior myocardial infarction, cerebrovascular disease, chronic kidney disease, diabetes, and others shown in Table 3. Medications associated with documented long-COVID care included opioids (AOR, 1.24; 95% CI, 1.17-1.30) and calcium channel blockers (AOR, 1.24; 95% CI, 1.20-1.27) but not antidepressants, angiotensin receptor blockers, angiotensin-converting enzyme inhibitors, or statins.

The number of primary care visits in the 2-year period before infection was not associated with long-COVID care (Table 3). However, the number of prior mental health visits was associated with long-COVID care, along with the number of specialty visits, which had an even greater magnitude of association.

### Associations Between Acute SARS-CoV-2 Disease Severity or Vaccination and Long-COVID Care

Persons who were hospitalized (AOR, 2.60; 95% CI, 2.51-2.69) and those who underwent mechanical ventilation (AOR, 2.46; 95% CI, 2.26-2.69) for acute COVID-19 were more likely to have documented long-COVID care (Table 4). The number of symptoms documented at the time of acute infection was progressively associated with higher likelihood of long-COVID care (patients with ≥5 symptoms vs those with no symptoms, AOR, 1.71; 95% CI, 1.65-1.78). Acute symptoms associated with long-COVID care included abdominal pain, chills, having a cold, cough, diarrhea, dyspnea, fatigue, fever, headache, myalgia, nausea, rhinorrhea, loss of smell, and loss of taste but not sore throat or rhinorrhea (Table 4).

**Table 4.** Associations Between Indices of Severity of Acute SARS-CoV-2 Infection and the Documentation of COVID-19 *ICD-10* Codes 3 or More Months After Testing Positive for SARS-CoV-2 Infection Among VA Enrollees Who Tested Positive for SARS-CoV-2 Infection From February 2020 to April 2021 With Follow-up Extending to December 31, 2021

Characteristics	COVID-19 *ICD-10* codes documented ≥3 mo after infection, patients, No. (%) (N = 198 601)		OR (95% CI)	
	No (n = 171 856)	Yes (n = 26 745)	Crude	Adjusted^a^
Hospitalization within 30 d of infection				
No	156 711 (88.5)	20 448 (11.5)	1 [Reference]	1 [Reference]
Yes	15 145 (70.6)	6297 (29.4)	3.19 (3.08-3.29)	2.60 (2.51-2.69)
Mechanical ventilation for acute infection				
No	170 279 (86.8)	25 951 (13.2)	1 [Reference]	1 [Reference]
Yes	1577 (66.5)	794 (33.5)	3.30 (3.03-3.60)	2.46 (2.26-2.69)
Vaccine doses received at the time of infection, No.^b^				
0	51 882 (88.4)	6811 (11.6)	1 [Reference]	1 [Reference]
1	5138 (86.9)	772 (13.1)	1.14 (1.06-1.24)	1.03 (0.95-1.12)
2	2184 (89.3)	263 (10.7)	0.92 (0.81-1.05)	0.78 (0.68-0.90)
Symptoms at presentation with acute infection, No.				
0	89 534 (89.6)	10 386 (10.4)	1 [Reference]	1 [Reference]
1-2	35 741 (84.3)	6675 (15.7)	1.61 (1.56-1.66)	1.46 (1.42-1.52)
3-4	24 304 (82.4)	5203 (17.6)	1.85 (1.78-1.91)	1.70 (1.64-1.76)
≥5	22 276 (83.3)	4481 (16.7)	1.73 (1.67-1.80)	1.71 (1.65-1.78)
Symptoms at the time of acute infection				
Abdominal pain				
No	167 301 (86.7)	25 668 (13.3)	1 [Reference]	1 [Reference]
Yes	4554 (80.9)	1077 (19.1)	1.54 (1.44-1.65)	1.31 (1.22-1.40)
Chills				
No	169 530 (86.6)	26 267 (13.4)	1 [Reference]	1 [Reference]
Yes	2325 (82.9)	478 (17.1)	1.33 (1.20-1.47)	1.33 (1.21-1.48)
Cold				
No	127 686 (87.2)	18 663 (12.8)	1 [Reference]	1 [Reference]
Yes	44 169 (84.5)	8082 (15.5)	1.25 (1.22-1.29)	1.28 (1.25-1.32)
Cough				
No	124 865 (88.0)	16 958 (12.0)	1 [Reference]	1 [Reference]
Yes	46 990 (82.8)	9787 (17.2)	1.53 (1.49-1.58)	1.48 (1.44-1.52)
Diarrhea				
No	154 403 (87.0)	23 086 (13.0)	1 [Reference]	1 [Reference]
Yes	17 452 (82.7)	3659 (17.3)	1.40 (1.35-1.46)	1.31 (1.26-1.37)
Dyspnea				
No	128 876 (88.3)	17 056 (11.7)	1 [Reference]	1 [Reference]
Yes	42 979 (81.6)	9689 (18.4)	1.70 (1.66-1.75)	1.60 (1.56-1.65)
Fatigue				
No	163 080 (87.0)	24 324 (13.0)	1 [Reference]	1 [Reference]
Yes	8775 (78.4)	2421 (21.6)	1.85 (1.76-1.94)	1.51 (1.44-1.59)
Fever				
No	131 565 (88.0)	17 957 (12.0)	1 [Reference]	1 [Reference]
Yes	40 290 (82.1)	8788 (17.9)	1.60 (1.55-1.64)	1.49 (1.45-1.54)
Headache				
No	149 664 (86.7)	22 925 (13.3)	1 [Reference]	1 [Reference]
Yes	22 191 (85.3)	3820 (14.7)	1.12 (1.08-1.17)	1.22 (1.17-1.27)
Loss of smell				
No	162 558 (86.5)	25 394 (13.5)	1 [Reference]	1 [Reference]
Yes	9297 (87.3)	1351 (12.7)	0.93 (0.88-0.99)	1.07 (1.01-1.14)
Loss of taste				
No	161 300 (86.5)	25 087 (13.5)	1 [Reference]	1 [Reference]
Yes	10 555 (86.4)	1658 (13.6)	1.01 (0.96-1.07)	1.12 (1.06-1.18)
Myalgia				
No	169 445 (86.6)	26 259 (13.4)	1 [Reference]	1 [Reference]
Yes	2410 (83.2)	486 (16.8)	1.30 (1.18-1.44)	1.36 (1.23-1.51)
Nausea				
No	159 223 (87.0)	23 857 (13.0)	1 [Reference]	1 [Reference]
Yes	12 632 (81.4)	2888 (18.6)	1.53 (1.46-1.59)	1.45 (1.39-1.51)
Rhinorrhea				
No	171 600 (86.5)	26 700 (13.5)	1 [Reference]	1 [Reference]
Yes	255 (85.0)	45 (15.0)	1.13 (0.83-1.56)	1.15 (0.83-1.59)
Sore throat				
No	166 067 (86.5)	25 860 (13.5)	1 [Reference]	1 [Reference]
Yes	5788 (86.7)	885 (13.3)	0.98 (0.91-1.06)	1.05 (0.97-1.13)

Abbreviations: *ICD-10, International Statistical Classification of Diseases and Related Health Problems, Tenth Revision*; OR, odds ratio; VA, Veterans Affairs Health Care System.

^a^ Adjusted by multivariable logistic regression for age (using the categories shown in Table 3), sex, race, ethnicity, urban vs rural residence, Charlson Comorbidity Index score, VA Integrated Service Network, time period of infection (categorized according to the waves of the pandemic as shown in Table 3), and number of primary care, mental health and specialty care encounters in the 2 years before infection.

^b^ When looking at COVID-19 vaccination, we limited analyses to persons infected after January 1, 2021, when vaccines became widely available, and adjusted for time of infection in monthly time periods, to account for rapid changes in vaccination status that occurred in the VA after January.

Persons who had received both doses of mRNA vaccine at the time of SARS-CoV-2 infection (2447 individuals) were less likely to have long-COVID care (AOR, 0.78; 95% CI, 0.68-0.90) than unvaccinated persons. However, persons who had received only a single dose of mRNA vaccination at the time of SARS-CoV-2 infection (5910 individuals) were not less likely to have long-COVID care (AOR, 1.03; 95% CI, 0.95-1.10) than unvaccinated persons (58 693 individuals).

### Associations With Follow-up Restricted to 3 to 8 Months From SARS-CoV-2 Infection

Long-COVID codes were documented in 8.9% of individuals (17 731 of 198 601 individuals), when follow-up extended only from 3 to 8 months (eTable 2 in the Supplement). There were only minor differences in the magnitude of the associations with follow-up extending from 3 to 8 months compared with follow-up extending to December 31, 2021.

## Discussion

In this cohort study of 198 601 survivors of acute SARS-CoV-2 infection in the VA health care system, 13.5% had documented COVID-19–related care 3 or more months after acute infection, delivered in a variety of clinical settings, with great variability across regions and medical centers. Factors independently associated with documentation of long-COVID care included older age, Black or American Indian/Alaska Native race (vs White race), Hispanic ethnicity, geographic region, high comorbidity burden, symptomatic acute presentation, hospitalization for acute presentation, and being unvaccinated at the time of infection.

There are numerous reports of approaches individual systems have taken to providing long-COVID care in specialized clinics.^19,20,21^ Such centers of excellence do not yet appear to exist at a scale that could provide care for all COVID-19 sequelae. We found large differences across the VA’s administrative regions in long-COVID care, ranging from 10.8% to 18.1% and even greater differences by medical center, ranging from 3.0% to 41.0% (Figure). Receipt of long-COVID care was documented in a wide variety of clinics, reflecting both the broad range of long-COVID manifestations, as well as the lack of specific stop-codes for dedicated long-COVID clinics. Although the VA has launched outreach and care networks for long COVID,^22^ including setting up specialized, multidisciplinary long-COVID clinics at multiple facilities, our data suggest that there is still wide variability in practice across the country in the evaluation and management of patients potentially experiencing long COVID.

Investigations of rates and risk factors for long COVID are hindered by lack of a universally accepted and validated definition of long COVID. Symptom-based approaches, such as that recommended the World Health Organization,^1^ are difficult to operationalize (eg, because of the lack of alternative diagnosis) and are likely to be modified over time. We investigated the factors associated with documentation of COVID-19–related *ICD-10 *codes more than 3 months after acute infection as a way of evaluating factors associated with health care encounters related to long COVID. This approach only captures symptoms and manifestations that were both reported by the patients to their practitioners and documented by the practitioners as being related to COVID-19 using *ICD-10* codes. Therefore, our approach underestimates the true prevalence of long-COVID symptoms and likely captures the subset of patients with more severe symptoms or manifestations of long COVID and their risk factors. Indeed, a systematic review^23^ of 57 studies including 250 351 survivors, most of whom (79%) were hospitalized for acute COVID-19, reported that 54% experienced at least 1 postacute sequelae of COVID-19 at 6 or more months after infection, which is much higher than the proportions we report. Small, single-center studies^8,9,24,25,26^ limited to hospitalized patients with follow-up of only 1 to 6 months reported a prevalence of long-COVID symptoms ranging from 32.6% to 87.4%, which is higher than the proportion we found of hospitalized patients who had documented long-COVID codes (29.3%). A large population-based study from England (the REACT-2 study)^27^ reported long-COVID symptoms lasting 12 or more weeks in 38.0% of patients (with at least 1 symptom) or 14.8% of patients (with at least 3 symptoms).

Early reports of long COVID were disseminated through social media platforms such as Twitter and Facebook. Long COVID may be the first illness in history that has been defined by patients through social media.^28^ This created misconceptions as to who is at risk for long COVID confounded by the characteristics of social media users. We found that patients who had more symptomatic acute disease or required hospitalization or ventilation were more likely to have documented long-COVID care. This suggests that although persons with asymptomatic or minimally symptomatic acute infection can certainly develop long COVID, those with more severe acute presentation are at much higher risk of requiring long-COVID care. This conclusion is consistent with a study^29^ of 4184 users of a COVID Symptom Study app, for whom experiencing more than 5 symptoms during the first week of illness was associated with self-reported long-COVID symptoms after 12 weeks. Other studies^8,13,30,31^ also suggested that that severity of acute COVID-19 illness (measured, for example, by admission to an intensive care unit or requirement for noninvasive or invasive ventilation) was associated with persistence of symptoms (eg, dyspnea, fatigue, muscular weakness, and posttraumatic stress disorder), reduction in health-related quality-of-life scores, pulmonary function abnormalities, and radiographic abnormalities in the postacute COVID-19 setting. We also found that the presence of multiple chronic conditions, as measured by the CCI score, was one of the factors most associated with risk of documented long-COVID care, as well as many individual conditions, such as COPD, asthma, cerebrovascular disease, cardiovascular disease, and chronic kidney disease. These findings suggest that although persons without chronic conditions can certainly develop long COVID, those with multiple chronic conditions are at much higher risk.

The associations we describe between racial and ethnic minoritized groups and documented long-COVID care are relatively novel. Black, American Indian/Alaska Native, and Hispanic people not only appear to have higher risk of acquiring COVID-19 and experiencing acute adverse outcomes, as described elsewhere,^32,33,34^ but also appear to be more likely to experience long COVID. These disparities may be even more pronounced in racial and ethnic minoritized groups that do not have access to comprehensive health care as provided by the VA health care system.

Emerging data appear to favor a potential protective effect of COVID-19 vaccination against developing long COVID symptoms or manifestations.^35,36,37,38,39^ Our data support this by demonstrating that persons who had received both doses of mRNA vaccine at the time of SARS-CoV-2 infection (ie, were considered fully vaccinated) were less likely to have received long-COVID care (AOR, 0.78; 95% CI, 0.68-0.90) than unvaccinated persons. We used receipt of a single dose of mRNA vaccination as a negative exposure control. The lack of association between receipt of a single vaccine dose and long-COVID care argues against the presence of residual confounding in our analyses.

### Limitations

This study has limitations that should be addressed. It is unclear what symptoms or manifestations might have prompted physicians to document a COVID-19–related *ICD-10* code more than 3 months after infection onset. However, because there is diagnostic uncertainty as to the nature of long COVID, evaluating risk factors for long COVID using the approach we selected without imposing a predetermined definition may actually be preferable. Persons more likely to have health care encounters for other, non–COVID-related conditions would be more likely to have long-COVID codes documented during follow-up. However, we adjusted for the number of encounters with primary, mental health, and specialty care before the infection to account for the propensity to have non–COVID-related encounters after the infection. It would be inappropriate to adjust for number of encounters after infection because those encounters may actually be caused by persistent COVID-related symptoms.

## Conclusions

Long-COVID care was documented in a variety of clinical settings, with great variability across regions and medical centers. Our findings of rates, clinical settings, and factors associated with long-COVID care provide support and guidance for health care systems to develop systematic approaches to the evaluation and management of patients who may be experiencing long COVID.

## Notes

Supplement. **eTable 1.** Description of the Multivariable Logistic Regression Models Used to Evaluate Factors Associated With the Outcome of Documentation of Long-COVID Care (ie, COVID-19 *ICD-10* Codes ≥3 Months After Testing Positive for SARS-CoV-2 Infection) Among 198,601 VA Enrollees Who Tested Positive for SARS-CoV-2 Infection From February 2020 to April 2021 With Follow-up Extending to December 31, 2021 **eTable 2.** Associations Between Baseline Characteristics and the Documentation of COVID-19 *ICD-10* Codes ≥3 Months After Testing Positive for SARS-CoV-2 Infection Among 198,601 VA Enrollees Who Tested Positive for SARS-CoV-2 Infection From February 2020 to April 2021 With Follow-up Extending From 90 to 240 Days Since Infection **eFigure.** Forest Plot of the Associations (Adjusted Odds Ratios) of Selected Patient Characteristics With Documentation of COVID-19* ICD-10* Codes ≥3 Months After Testing Positive for SARS-CoV-2 Infection Among 198,601 VA Enrollees Who Tested Positive for SARS-CoV-2 Infection From February 2020 to April 2021 With Follow-up Extending to December 31, 2021
