# Supplementary material for: Rates and Factors Associated With Documentation of Diagnostic Codes for Long COVID in the National Veterans Affairs Health Care System
Source: JAMA Netw Open. 2022 Jul 29;5(7):e2224359. doi: 10.1001/jamanetworkopen.2022.24359 (PMC9338411; doi:10.1001/jamanetworkopen.2022.24359)
Supplement: Supplement. — eTable 1. Description of the Multivariable Logistic Regression Models Used to Evaluate Factors Associated With the Outcome of Documentation of Long-COVID Care (ie, COVID-19 ICD-10 Codes ≥3 Months After Testing Positive for SARS-CoV-2 Infection) Among 198,601 VA Enrollees Who Tested Positive for SARS-CoV-2 Infection From February 2020 to April 2021 With Follow-up Extending to December 31, 2021 eTable 2. Associations Between Baseline Characteristics and the Documentation of COVID-19 ICD-10 Codes ≥3 Months After Testing Positive for SARS-CoV-2 Infection Among 198,601 VA Enrollees Who Tested Positive for SARS-CoV-2 Infection From February 2020 to April 2021 With Follow-up Extending From 90 to 240 Days Since Infection eFigure. Forest Plot of the Associations (Adjusted Odds Ratios) of Selected Patient Characteristics With Documentation of COVID-19 ICD-10 Codes ≥3 Months After Testing Positive for SARS-CoV-2 Infection Among 198,601 VA Enrollees Who Tested Positive for SARS-CoV-2 Infection From February 2020 to April 2021 With Follow-up Extending to December 31, 2021 [file jamanetwopen-e2224359-s001.pdf]

Supplemental Online Content

Ioannou GN, Baraff A, Fox A, et al. Rates and factors associated with documentation of diagnostic codes for long COVID in the national Veterans Affairs health care system. *JAMA Netw Open*. 2022;5(7):e2224359. doi:10.1001/jamanetworkopen.2022.24359

eTable 1. Description of the Multivariable Logistic Regression Models Used to Evaluate Factors Associated With the Outcome of Documentation of Long-COVID Care (ie, COVID-19 *ICD-10* Codes ≥3 Months After Testing Positive for SARS-CoV-2 Infection) Among 198,601 VA Enrollees Who Tested Positive for SARS-CoV-2 Infection From February 2020 to April 2021 With Follow-up Extending to December 31, 2021
eTable 2. Associations Between Baseline Characteristics and the Documentation of COVID-19 *ICD-10* Codes ≥3 Months After Testing Positive for SARS-CoV-2 Infection Among 198,601 VA Enrollees Who Tested Positive for SARS-CoV-2 Infection From February 2020 to April 2021 With Follow-up Extending From 90 to 240 Days Since Infection
eFigure. Forest Plot of the Associations (Adjusted Odds Ratios) of Selected Patient Characteristics With Documentation of COVID-19 *ICD-10* Codes ≥3 Months After Testing Positive for SARS-CoV-2 Infection Among 198,601 VA Enrollees Who Tested Positive for SARS-CoV-2 Infection From February 2020 to April 2021 With Follow-up Extending to December 31, 2021

This supplemental material has been provided by the authors to give readers additional information about their work.

© 2022 Ioannou GN et al. *JAMA Network Open.*

eTable 1. Description of the Multivariable Logistic Regression Models Used to Evaluate Factors Associated With the Outcome of Documentation of Long-COVID Care (ie, COVID-19 *ICD-10* Codes ≥3 Months After Testing Positive for SARS-CoV-2 Infection) Among 198,601 VA Enrollees Who Tested Positive for SARS-CoV-2 Infection From February 2020 to April 2021 With Follow-up Extending to December 31, 2021

**Characteristic of Interest**	**Co-variates included in the logistic regression model**	**Study population: Time Period of SARS-CoV-2 Infection**	**Follow-up period for outcome ascertainment**
**Age**	Age, sex, race, ethnicity, urban/rural residence, VISN*, time period of infection, number of primary care visits in the prior 2 years, number of mental health visits in the prior 2 years and number of specialty care visits in the prior 2 years and **Charlson Comorbidity Index**	February 1, 2020 to April 30, 2021	Extending to December 31, 2021
**Sex**	Age, sex, race, ethnicity, urban/rural residence, VISN*, time period of infection, number of primary care visits in the prior 2 years, number of mental health visits in the prior 2 years and number of specialty care visits in the prior 2 years and **Charlson Comorbidity Index**	February 1, 2020 to April 30, 2021	Extending to December 31, 2021
**Race**	Age, sex, race, ethnicity, urban/rural residence, VISN*, time period of infection, number of primary care visits in the prior 2 years, number of mental health visits in the prior 2 years and number of specialty care visits in the prior 2 years and **Charlson Comorbidity Index**	February 1, 2020 to April 30, 2021	Extending to December 31, 2021
**Ethnicity**	Age, sex, race, ethnicity, urban/rural residence, VISN*, time period of infection, number of primary care visits in the prior 2 years, number of mental health visits in the prior 2 years and number of specialty care visits in the prior 2 years and **Charlson Comorbidity Index**	February 1, 2020 to April 30, 2021	Extending to December 31, 2021
**urban/rural residence**	Age, sex, race, ethnicity, urban/rural residence, VISN*, time period of infection, number of primary care visits in the prior 2 years, number of mental health visits in the prior 2 years and number of specialty care visits in the prior 2 years and **Charlson Comorbidity Index**	February 1, 2020 to April 30, 2021	Extending to December 31, 2021

© 2022 Ioannou GN et al. *JAMA Network Open.*

**Charlson Comorbidity Index (CCI)**	Age, sex, race, ethnicity, urban/rural residence, VISN*, time period of infection, number of primary care visits in the prior 2 years, number of mental health visits in the prior 2 years and number of specialty care visits in the prior 2 years and **Charlson Comorbidity Index**	February 1, 2020 to April 30, 2021	Extending to December 31, 2021
**VISN**	Age, sex, race, ethnicity, urban/rural residence, VISN*, time period of infection, number of primary care visits in the prior 2 years, number of mental health visits in the prior 2 years and number of specialty care visits in the prior 2 years and **Charlson Comorbidity Index**	February 1, 2020 to April 30, 2021	Extending to December 31, 2021
**Time period of infection**	Age, sex, race, ethnicity, urban/rural residence, VISN*, time period of infection, number of primary care visits in the prior 2 years, number of mental health visits in the prior 2 years and number of specialty care visits in the prior 2 years and **Charlson Comorbidity Index**	February 1, 2020 to April 30, 2021	Extending up to 8 months after infection
**BMI**	Age, sex, race, ethnicity, urban/rural residence, VISN*, time period of infection, number of primary care visits in the prior 2 years, number of mental health visits in the prior 2 years and number of specialty care visits in the prior 2 years and **Charlson Comorbidity Index and BMI**	February 1, 2020 to April 30, 2021	Extending to December 31, 2021
**Diabetes**	Age, sex, race, ethnicity, urban/rural residence, VISN*, time period of infection, number of primary care visits in the prior 2 years, number of mental health visits in the prior 2 years and number of specialty care visits in the prior 2 years and **Diabetes**	February 1, 2020 to April 30, 2021	Extending to December 31, 2021
**Chronic Obstructive Pulmonary Disease (COPD)**	Age, sex, race, ethnicity, urban/rural residence, VISN*, time period of infection, number of primary care visits in the prior 2 years, number of mental health visits in the prior 2 years and number of specialty care visits in the prior 2 years and **COPD**	February 1, 2020 to April 30, 2021	Extending to December 31, 2021
**Asthma**	Age, sex, race, ethnicity, urban/rural residence, VISN*, time period of infection, number of primary care visits in the prior 2 years, number of mental health visits in the prior 2 years and	February 1, 2020 to April 30, 2021	Extending to December 31, 2021

© 2022 Ioannou GN et al. *JAMA Network Open.*

	number of specialty care visits in the prior 2 years and **Asthma**		
**Congestive Heart Failure (CHF)**	Age, sex, race, ethnicity, urban/rural residence, VISN*, time period of infection, number of primary care visits in the prior 2 years, number of mental health visits in the prior 2 years and number of specialty care visits in the prior 2 years and **CHF**	February 1, 2020 to April 30, 2021	Extending to December 31, 2021
**Myocardial Infarction (MI)**	Age, sex, race, ethnicity, urban/rural residence, VISN*, time period of infection, number of primary care visits in the prior 2 years, number of mental health visits in the prior 2 years and number of specialty care visits in the prior 2 years and **MI**	February 1, 2020 to April 30, 2021	Extending to December 31, 2021
**Cerebrovascular Disease**	Age, sex, race, ethnicity, urban/rural residence, VISN*, time period of infection, number of primary care visits in the prior 2 years, number of mental health visits in the prior 2 years and number of specialty care visits in the prior 2 years and **Cerebrovascular Disease**	February 1, 2020 to April 30, 2021	Extending to December 31, 2021
**Chronic Kidney Disease**	Age, sex, race, ethnicity, urban/rural residence, VISN*, time period of infection, number of primary care visits in the prior 2 years, number of mental health visits in the prior 2 years and number of specialty care visits in the prior 2 years and **Chronic Kidney Disease**	February 1, 2020 to April 30, 2021	Extending to December 31, 2021
**Peripheral Arterial Disease**	Age, sex, race, ethnicity, urban/rural residence, VISN*, time period of infection, number of primary care visits in the prior 2 years, number of mental health visits in the prior 2 years and number of specialty care visits in the prior 2 years and **Peripheral Arterial Disease**	February 1, 2020 to April 30, 2021	Extending to December 31, 2021
**Venous Thromboembolism**	Age, sex, race, ethnicity, urban/rural residence, VISN*, time period of infection, number of primary care visits in the prior 2 years, number of mental health visits in the prior 2 years and number of specialty care visits in the prior 2 years and **Venous Thromboembolism**	February 1, 2020 to April 30, 2021	Extending to December 31, 2021
**Obstructive Sleep Apnea**	Age, sex, race, ethnicity, urban/rural residence, VISN*, time period of infection, number of primary care visits in the prior 2 years, number of mental	February 1, 2020 to April 30, 2021	Extending to December 31, 2021

© 2022 Ioannou GN et al. *JAMA Network Open.*

	health visits in the prior 2 years and number of specialty care visits in the prior 2 years and **Obstructive Sleep Apnea**		
**Obesity Hypoventilation Syndrome**	Age, sex, race, ethnicity, urban/rural residence, VISN*, time period of infection, number of primary care visits in the prior 2 years, number of mental health visits in the prior 2 years and number of specialty care visits in the prior 2 years and **Obesity Hypoventilation Syndrome**	February 1, 2020 to April 30, 2021	Extending to December 31, 2021
**Opioids**	Age, sex, race, ethnicity, urban/rural residence, VISN*, time period of infection, number of primary care visits in the prior 2 years, number of mental health visits in the prior 2 years and number of specialty care visits in the prior 2 years and **Charlson Comorbidity Index and Opioids**	February 1, 2020 to April 30, 2021	Extending to December 31, 2021
**Antidepressants**	Age, sex, race, ethnicity, urban/rural residence, VISN*, time period of infection, number of primary care visits in the prior 2 years, number of mental health visits in the prior 2 years and number of specialty care visits in the prior 2 years and **Charlson Comorbidity Index and Antidepressants**	February 1, 2020 to April 30, 2021	Extending to December 31, 2021
**Statins**	Age, sex, race, ethnicity, urban/rural residence, VISN*, time period of infection, number of primary care visits in the prior 2 years, number of mental health visits in the prior 2 years and number of specialty care visits in the prior 2 years and **Charlson Comorbidity Index and Statins**	February 1, 2020 to April 30, 2021	Extending to December 31, 2021
**ACE Inhibitors**	Age, sex, race, ethnicity, urban/rural residence, VISN*, time period of infection, number of primary care visits in the prior 2 years, number of mental health visits in the prior 2 years and number of specialty care visits in the prior 2 years and **Charlson Comorbidity Index and ACE Inhibitors**	February 1, 2020 to April 30, 2021	Extending to December 31, 2021
**Angiotensin receptor blockers (ARBs)**	Age, sex, race, ethnicity, urban/rural residence, VISN*, time period of infection, number of primary care visits in the prior 2 years, number of mental health visits in the prior 2 years and number of specialty care visits in the prior 2 years and **Charlson Comorbidity**	February 1, 2020 to April 30, 2021	Extending to December 31, 2021

© 2022 Ioannou GN et al. *JAMA Network Open.*

	**Index and Angiotensin receptor blockers**		
**Calcium Channel Blockers**	Age, sex, race, ethnicity, urban/rural residence, VISN*, time period of infection, number of primary care visits in the prior 2 years, number of mental health visits in the prior 2 years and number of specialty care visits in the prior 2 years and **Charlson Comorbidity Index and calcium channel blockers**	February 1, 2020 to April 30, 2021	Extending to December 31, 2021
**Number of primary care visits in prior 2 years**	Age, sex, race, ethnicity, urban/rural residence, VISN*, time period of infection, number of primary care visits in the prior 2 years, number of mental health visits in the prior 2 years and number of specialty care visits in the prior 2 years and **Charlson Comorbidity Index**	February 1, 2020 to April 30, 2021	Extending to December 31, 2021
**Number of mental health visits in prior 2 years**	Age, sex, race, ethnicity, urban/rural residence, VISN*, time period of infection, number of primary care visits in the prior 2 years, number of mental health visits in the prior 2 years and number of specialty care visits in the prior 2 years and **Charlson Comorbidity Index**	February 1, 2020 to April 30, 2021	Extending to December 31, 2021
**Number of specialty care visits in prior 2 years**	Age, sex, race, ethnicity, urban/rural residence, VISN*, time period of infection, number of primary care visits in the prior 2 years, number of mental health visits in the prior 2 years and number of specialty care visits in the prior 2 years and **Charlson Comorbidity Index**	February 1, 2020 to April 30, 2021	Extending to December 31, 2021
**Hospitalization within 30 days of infection**	Age, sex, race, ethnicity, urban/rural residence, VISN*, time period of infection, number of primary care visits in the prior 2 years, number of mental health visits in the prior 2 years and number of specialty care visits in the prior 2 years and **Charlson Comorbidity Index**	February 1, 2020 to April 30, 2021	Extending to December 31, 2021
**Mechanical Ventilation for acute infection**	Age, sex, race, ethnicity, urban/rural residence, VISN*, time period of infection, number of primary care visits in the prior 2 years, number of mental health visits in the prior 2 years and number of specialty care visits in the prior 2 years and **Charlson Comorbidity Index**	February 1, 2020 to April 30, 2021	Extending to December 31, 2021

© 2022 Ioannou GN et al. *JAMA Network Open.*

**Number of vaccine doses received at the time of infection†**	Age, sex, race, ethnicity, urban/rural residence, VISN*, time period of infection, number of primary care visits in the prior 2 years, number of mental health visits in the prior 2 years and number of specialty care visits in the prior 2 years and **Charlson Comorbidity Index**	January 1, 2021 to April 30, 2021	Extending to December 31, 2021
**Number of Symptoms at presentation with acute infection**	Age, sex, race, ethnicity, urban/rural residence, VISN*, time period of infection, number of primary care visits in the prior 2 years, number of mental health visits in the prior 2 years and number of specialty care visits in the prior 2 years and **Charlson Comorbidity Index**	February 1, 2020 to April 30, 2021	Extending to December 31, 2021
**Symptoms at the time of acute infection**	Age, sex, race, ethnicity, urban/rural residence, VISN*, time period of infection, number of primary care visits in the prior 2 years, number of mental health visits in the prior 2 years and number of specialty care visits in the prior 2 years and **Charlson Comorbidity Index**	February 1, 2020 to April 30, 2021	Extending to December 31, 2021
**Abdominal pain**	Age, sex, race, ethnicity, urban/rural residence, VISN*, time period of infection, number of primary care visits in the prior 2 years, number of mental health visits in the prior 2 years and number of specialty care visits in the prior 2 years and **Charlson Comorbidity Index**	February 1, 2020 to April 30, 2021	Extending to December 31, 2021
**Chills**	Age, sex, race, ethnicity, urban/rural residence, VISN*, time period of infection, number of primary care visits in the prior 2 years, number of mental health visits in the prior 2 years and number of specialty care visits in the prior 2 years and **Charlson Comorbidity Index**	February 1, 2020 to April 30, 2021	Extending to December 31, 2021
**Cold**	Age, sex, race, ethnicity, urban/rural residence, VISN*, time period of infection, number of primary care visits in the prior 2 years, number of mental health visits in the prior 2 years and number of specialty care visits in the prior 2 years and **Charlson Comorbidity Index**	February 1, 2020 to April 30, 2021	Extending to December 31, 2021
**Cough**	Age, sex, race, ethnicity, urban/rural residence, VISN*, time period of infection, number of primary care visits in the prior 2 years, number of mental	February 1, 2020 to April 30, 2021	Extending to December 31, 2021

© 2022 Ioannou GN et al. *JAMA Network Open.*

	health visits in the prior 2 years and number of specialty care visits in the prior 2 years and **Charlson Comorbidity Index**		
**Diarrhea**	Age, sex, race, ethnicity, urban/rural residence, VISN*, time period of infection, number of primary care visits in the prior 2 years, number of mental health visits in the prior 2 years and number of specialty care visits in the prior 2 years and **Charlson Comorbidity Index**	February 1, 2020 to April 30, 2021	Extending to December 31, 2021
**Dyspnea**	Age, sex, race, ethnicity, urban/rural residence, VISN*, time period of infection, number of primary care visits in the prior 2 years, number of mental health visits in the prior 2 years and number of specialty care visits in the prior 2 years and **Charlson Comorbidity Index**	February 1, 2020 to April 30, 2021	Extending to December 31, 2021
**Fatigue**	Age, sex, race, ethnicity, urban/rural residence, VISN*, time period of infection, number of primary care visits in the prior 2 years, number of mental health visits in the prior 2 years and number of specialty care visits in the prior 2 years and **Charlson Comorbidity Index**	February 1, 2020 to April 30, 2021	Extending to December 31, 2021
**Fever**	Age, sex, race, ethnicity, urban/rural residence, VISN*, time period of infection, number of primary care visits in the prior 2 years, number of mental health visits in the prior 2 years and number of specialty care visits in the prior 2 years and **Charlson Comorbidity Index**	February 1, 2020 to April 30, 2021	Extending to December 31, 2021
**Headache**	Age, sex, race, ethnicity, urban/rural residence, VISN*, time period of infection, number of primary care visits in the prior 2 years, number of mental health visits in the prior 2 years and number of specialty care visits in the prior 2 years and **Charlson Comorbidity Index**	February 1, 2020 to April 30, 2021	Extending to December 31, 2021
**Loss of smell**	Age, sex, race, ethnicity, urban/rural residence, VISN*, time period of infection, number of primary care visits in the prior 2 years, number of mental health visits in the prior 2 years and number of specialty care visits in the prior 2 years and **Charlson Comorbidity Index**	February 1, 2020 to April 30, 2021	Extending to December 31, 2021

© 2022 Ioannou GN et al. *JAMA Network Open.*

**Loss of taste**	Age, sex, race, ethnicity, urban/rural residence, VISN*, time period of infection, number of primary care visits in the prior 2 years, number of mental health visits in the prior 2 years and number of specialty care visits in the prior 2 years and **Charlson Comorbidity Index**	February 1, 2020 to April 30, 2021	Extending to December 31, 2021
**Myalgia**	Age, sex, race, ethnicity, urban/rural residence, VISN*, time period of infection, number of primary care visits in the prior 2 years, number of mental health visits in the prior 2 years and number of specialty care visits in the prior 2 years and **Charlson Comorbidity Index**	February 1, 2020 to April 30, 2021	Extending to December 31, 2021
**Nausea**	Age, sex, race, ethnicity, urban/rural residence, VISN*, time period of infection, number of primary care visits in the prior 2 years, number of mental health visits in the prior 2 years and number of specialty care visits in the prior 2 years and **Charlson Comorbidity Index**	February 1, 2020 to April 30, 2021	Extending to December 31, 2021
**Rhinorrhea**	Age, sex, race, ethnicity, urban/rural residence, VISN*, time period of infection, number of primary care visits in the prior 2 years, number of mental health visits in the prior 2 years and number of specialty care visits in the prior 2 years and **Charlson Comorbidity Index**	February 1, 2020 to April 30, 2021	Extending to December 31, 2021
**Sore throat**	Age, sex, race, ethnicity, urban/rural residence, VISN*, time period of infection, number of primary care visits in the prior 2 years, number of mental health visits in the prior 2 years and number of specialty care visits in the prior 2 years and **Charlson Comorbidity Index**	February 1, 2020 to April 30, 2021	Extending to December 31, 2021

* VISN is the VA Integrated Service Network

© 2022 Ioannou GN et al. *JAMA Network Open.*

eTable 2. Associations Between Baseline Characteristics and the Documentation of COVID-19 *ICD-10* Codes ≥3 Months After Testing Positive for SARS-CoV-2 Infection Among 198,601 VA Enrollees Who Tested Positive for SARS-CoV-2 Infection From February 2020 to April 2021 With Follow-up Extending From 90 to 240 Days Since Infection

	**COVID-19 ICD-10 codes documented from 90 to 240 days after infection, N (%)**			
**Characteristics**	**No N=180,870**	**Yes N=17,731**	**Crude Odds Ratio**	**Adjusted Odds Ratio***
**A. Sociodemographic characteristics**				
**Age, years**				
18-49	49125 (94.1%)	3098 (5.9%)	1	1
50-69	33343 (91.4%)	3142 (8.6%)	1.49 (1.42, 1.57)	1.33 (1.26, 1.41)
60-64	18884 (90.7%)	1935 (9.3%)	1.62 (1.53, 1.72)	1.34 (1.25, 1.42)
65-69	18497 (89.5%)	2174 (10.5%)	1.86 (1.76, 1.97)	1.46 (1.37, 1.56)
70-74	31325 (89.4%)	3724 (10.6%)	1.89 (1.79, 1.98)	1.49 (1.40, 1.58)
75-79	14669 (88.8%)	1844 (11.2%)	1.99 (1.88, 2.12)	1.56 (1.46, 1.67)
80-84	6901 (88.9%)	858 (11.1%)	1.97 (1.82, 2.14)	1.57 (1.44, 1.72)
85-89	5069 (89.3%)	606 (10.7%)	1.90 (1.73, 2.08)	1.55 (1.41, 1.71)
≥90	3045 (89.7%)	350 (10.3%)	1.82 (1.62, 2.05)	1.53 (1.36, 1.73)
**Sex**				
Male	160963 (91.0%)	15979 (9.0%)	1	1
Female	19907 (91.9%)	1752 (8.1%)	0.89 (0.84, 0.93)	1.06 (1.01, 1.12)
**Race**				
White	122191 (91.2%)	11733 (8.8%)	1	1
African American or Black	40529 (90.6%)	4204 (9.4%)	1.08 (1.04, 1.12)	1.11 (1.06, 1.15)
Asian	1811 (92.2%)	153 (7.8%)	0.88 (0.75, 1.04)	1.13 (0.95, 1.34)
American Indian or Alaska Native	1650 (90.3%)	178 (9.7%)	1.12 (0.96, 1.31)	1.23 (1.05, 1.44)
Native Hawaiian or Pacific Islander	1702 (90.5%)	179 (9.5%)	1.10 (0.94, 1.28)	1.10 (0.94, 1.29)
Declined or Missing	12987 (91.0%)	1284 (9.0%)	1.03 (0.97, 1.09)	1.09 (1.02, 1.17)
**Ethnicity**				
Not Hispanic or Latino	156508 (91.1%)	15214 (8.9%)	1	1
Hispanic or Latino	17810 (90.2%)	1925 (9.8%)	1.11 (1.06, 1.17)	1.17 (1.10, 1.23)
Declined or Missing	6552 (91.7%)	592 (8.3%)	0.93 (0.85, 1.01)	0.94 (0.86, 1.04)
**Rural/Urban Residence**				
Rural	27932 (92.1%)	2394 (7.9%)	1	1
Urban	126030 (91.1%)	12252 (8.9%)	1.13 (1.08, 1.19)	1.12 (1.06, 1.17)
Unknown	26908 (89.7%)	3085 (10.3%)	1.34 (1.26, 1.41)	1.35 (1.27, 1.43)
**VA Integrated Service Network (VISN)**				
VISN 08	14081 (90.0%)	1571 (10.0%)	1	1
VISN 01	6229 (92.3%)	521 (7.7%)	0.75 (0.68, 0.83)	0.79 (0.71, 0.88)
VISN 02	8036 (91.3%)	768 (8.7%)	0.86 (0.78, 0.94)	0.88 (0.80, 0.97)
VISN 04	7975 (91.2%)	765 (8.8%)	0.86 (0.79, 0.94)	0.91 (0.83, 0.99)
VISN 05	4812 (88.5%)	626 (11.5%)	1.17 (1.06, 1.29)	1.18 (1.07, 1.30)
VISN 06	11300 (92.9%)	866 (7.1%)	0.69 (0.63, 0.75)	0.69 (0.63, 0.76)

© 2022 Ioannou GN et al. *JAMA Network Open.*

VISN 07	15480 (91.5%)	1429 (8.5%)	0.83 (0.77, 0.89)	0.88 (0.81, 0.95)
VISN 09	8424 (91.7%)	759 (8.3%)	0.81 (0.74, 0.88)	0.79 (0.72, 0.86)
VISN 10	14082 (90.7%)	1449 (9.3%)	0.92 (0.86, 0.99)	0.92 (0.85, 0.99)
VISN 12	8869 (91.1%)	870 (8.9%)	0.88 (0.81, 0.96)	0.89 (0.82, 0.98)
VISN 15	8746 (91.9%)	769 (8.1%)	0.79 (0.72, 0.86)	0.82 (0.75, 0.90)
VISN 16	12387 (92.4%)	1015 (7.6%)	0.73 (0.68, 0.80)	0.78 (0.72, 0.85)
VISN 17	12892 (86.7%)	1971 (13.3%)	1.37 (1.28, 1.47)	1.49 (1.39, 1.60)
VISN 19	8665 (90.8%)	881 (9.2%)	0.91 (0.84, 0.99)	1.01 (0.92, 1.10)
VISN 20	4636 (91.4%)	437 (8.6%)	0.84 (0.76, 0.94)	0.93 (0.84, 1.05)
VISN 21	7718 (90.6%)	798 (9.4%)	0.93 (0.85, 1.01)	0.96 (0.88, 1.05)
VISN 22	15786 (91.6%)	1445 (8.4%)	0.82 (0.76, 0.88)	0.85 (0.79, 0.92)
VISN 23	10749 (93.1%)	791 (6.9%)	0.66 (0.60, 0.72)	0.72 (0.66, 0.79)
**Time period of infection**				
Before June 1, 2020	10867 (93.5%)	757 (6.5%)	1	1
June 1 to October 31, 2020	43625 (91.3%)	4181 (8.7%)	1.38 (1.27, 1.49)	1.52 (1.40, 1.65)
November 1, 2020 to April 30, 2021	126378 (90.8%)	12793 (9.2%)	1.45 (1.35, 1.57)	1.65 (1.52, 1.78)
**B. Comorbid Conditions**				
**Charlson Comorbidity Index (CCI)**				
0	73521 (93.9%)	4817 (6.1%)	1	1
1	38055 (91.5%)	3548 (8.5%)	1.42 (1.36, 1.49)	1.24 (1.18, 1.30)
2	26940 (90.5%)	2844 (9.5%)	1.61 (1.54, 1.69)	1.29 (1.22, 1.36)
3	14911 (88.4%)	1962 (11.6%)	2.01 (1.90, 2.12)	1.49 (1.41, 1.59)
4	10289 (87.7%)	1441 (12.3%)	2.14 (2.01, 2.28)	1.52 (1.42, 1.63)
5-6	10706 (85.9%)	1752 (14.1%)	2.50 (2.36, 2.65)	1.70 (1.59, 1.82)
7-8	4460 (83.4%)	890 (16.6%)	3.05 (2.82, 3.29)	2.00 (1.84, 2.18)
≥9	1988 (80.6%)	477 (19.4%)	3.66 (3.30, 4.06)	2.30 (2.05, 2.57)
**Body Mass Index, Kg/m^2**				
<18.5	1438 (88.0%)	196 (12.0%)	1.23 (1.05, 1.43)	1.06 (0.91, 1.24)
18.5-25	25277 (90.0%)	2802 (10.0%)	1	1
>25-30	58395 (91.4%)	5490 (8.6%)	0.85 (0.81, 0.89)	0.91 (0.87, 0.96)
>30-35	52970 (91.5%)	4921 (8.5%)	0.84 (0.80, 0.88)	0.91 (0.87, 0.96)
>35-40	26611 (91.1%)	2614 (8.9%)	0.89 (0.84, 0.94)	0.96 (0.90, 1.01)
>40	15046 (90.0%)	1675 (10.0%)	1.00 (0.94, 1.07)	1.07 (1.00, 1.14)
**Diabetes**				
No	120270 (92.1%)	10357 (7.9%)	1	1
Yes	60599 (89.2%)	7374 (10.8%)	1.41 (1.37, 1.46)	1.08 (1.04, 1.11)
**Chronic Obstructive Pulmonary Disease**				
No	155169 (91.9%)	13662 (8.1%)	1	1
Yes	25700 (86.3%)	4069 (13.7%)	1.80 (1.73, 1.87)	1.42 (1.36, 1.48)
**Asthma**				
No	168131 (91.3%)	16034 (8.7%)	1	1
Yes	12738 (88.2%)	1697 (11.8%)	1.40 (1.32, 1.47)	1.32 (1.25, 1.39)

© 2022 Ioannou GN et al. *JAMA Network Open.*

**Congestive Heart Failure**				
No	169258 (91.5%)	15652 (8.5%)	1	1
Yes	11611 (84.8%)	2079 (15.2%)	1.94 (1.84, 2.03)	1.36 (1.29, 1.43)
**Myocardial Infarction**				
No	177345 (91.2%)	17111 (8.8%)	1	1
Yes	3524 (85.0%)	620 (15.0%)	1.82 (1.67, 1.99)	1.33 (1.22, 1.46)
**Cerebrovascular Disease**				
No	177667 (91.2%)	17202 (8.8%)	1	1
Yes	3202 (85.8%)	529 (14.2%)	1.71 (1.55, 1.87)	1.26 (1.15, 1.39)
**Chronic Kidney Disease**				
No	158427 (91.7%)	14374 (8.3%)	1	1
Yes	22442 (87.0%)	3357 (13.0%)	1.65 (1.58, 1.72)	1.23 (1.17, 1.28)
**Peripheral Arterial Disease**				
No	163641 (91.6%)	15014 (8.4%)	1	1
Yes	17228 (86.4%)	2717 (13.6%)	1.72 (1.65, 1.80)	1.24 (1.19, 1.30)
**Venous Thromboembolism**				
No	176715 (91.2%)	17020 (8.8%)	1	1
Yes	4154 (85.4%)	711 (14.6%)	1.78 (1.64, 1.93)	1.34 (1.24, 1.46)
**Obstructive Sleep Apnea**				
No	122371 (91.8%)	10929 (8.2%)	1	1
Yes	58498 (89.6%)	6802 (10.4%)	1.30 (1.26, 1.34)	1.15 (1.11, 1.19)
**Obesity Hypoventilation Syndrome**				
No	180112 (91.1%)	17574 (8.9%)	1	1
Yes	757 (82.8%)	157 (17.2%)	2.13 (1.79, 2.53)	1.57 (1.32, 1.87)
**C. Medications**				
**Opioids**				
No	171575 (91.4%)	16242 (8.6%)	1	1
Yes	9295 (86.2%)	1489 (13.8%)	1.69 (1.60, 1.79)	1.26 (1.19, 1.34)
**Antidepressants**				
No	122314 (91.5%)	11428 (8.5%)	1	1
Yes	58556 (90.3%)	6303 (9.7%)	1.15 (1.12, 1.19)	1.02 (0.98, 1.05)
**Statins**				
No	90490 (92.6%)	7193 (7.4%)	1	1
Yes	90380 (89.6%)	10538 (10.4%)	1.47 (1.42, 1.51)	1.00 (0.96, 1.04)
**ACE Inhibitors**				
No	128323 (91.7%)	11681 (8.3%)	1	1
Yes	52547 (89.7%)	6050 (10.3%)	1.26 (1.22, 1.31)	0.99 (0.95, 1.02)
**ARBs**				
No	155699 (91.5%)	14542 (8.5%)	1	1
Yes	25171 (88.8%)	3189 (11.2%)	1.36 (1.30, 1.41)	1.04 (0.99, 1.08)
**Calcium Channel Blockers**				
No	115061 (92.2%)	9697 (7.8%)	1	1

© 2022 Ioannou GN et al. *JAMA Network Open.*

Yes	65809 (89.1%)	8034 (10.9%)	1.45 (1.40, 1.49)	1.22 (1.18, 1.26)
**D. Healthcare Utilization**				
**Number of primary care visits in prior 2 years**				
0-5	87409 (92.9%)	6674 (7.1%)	1	1
6-11	50835 (90.8%)	5164 (9.2%)	1.33 (1.28, 1.38)	0.99 (0.95, 1.04)
≥12	41380 (87.7%)	5780 (12.3%)	1.83 (1.76, 1.90)	0.96 (0.90, 1.01)
**Number of mental health visits in prior 2 years**				
0	100656 (91.5%)	9401 (8.5%)	1	1
1-6	36020 (91.1%)	3535 (8.9%)	1.05 (1.01, 1.09)	1.02 (0.98, 1.07)
7-19	24854 (90.5%)	2607 (9.5%)	1.12 (1.07, 1.18)	1.10 (1.05, 1.15)
≥20	18094 (89.7%)	2075 (10.3%)	1.23 (1.17, 1.29)	1.15 (1.09, 1.21)
**Number of specialty care visits in prior 2 years**				
0	3553 (94.8%)	193 (5.2%)	1	1
1-9	86819 (93.4%)	6163 (6.6%)	1.31 (1.13, 1.51)	1.19 (1.03, 1.38)
10-18	47577 (90.8%)	4799 (9.2%)	1.86 (1.60, 2.15)	1.49 (1.28, 1.73)
≥19	41675 (86.6%)	6463 (13.4%)	2.85 (2.46, 3.31)	1.95 (1.67, 2.29)

** Adjusted by multivariable logistic regression for age (using the categories shown), sex, race, ethnicity, urban/rural residence, CCI, VISN, time period of infection (categorized according to the "waves" of the pandemic as shown),* and number of primary care, mental health and specialty care encounters in the two year prior to infection.
When we evaluated the associations of any of the individual comorbidities (e.g. COPD, CHF, CKD, diabetes, depression, PTSD, bipolar/schizoaffective, cancer, hypertension, obesity, cerebrovascular disease, smoking and others) we did not simultaneously adjust for the CCI because it would result in overadjustment.

© 2022 Ioannou GN et al. *JAMA Network Open.*

eFigure. Forest Plot of the Associations (Adjusted Odds Ratios) of Selected Patient Characteristics With Documentation of COVID-19 *ICD-10* Codes ≥3 Months After Testing Positive for SARS-CoV-2 Infection Among 198,601 VA Enrollees Who Tested Positive for SARS-CoV-2 Infection From February 2020 to April 2021 With Follow-up Extending to December 31, 2021

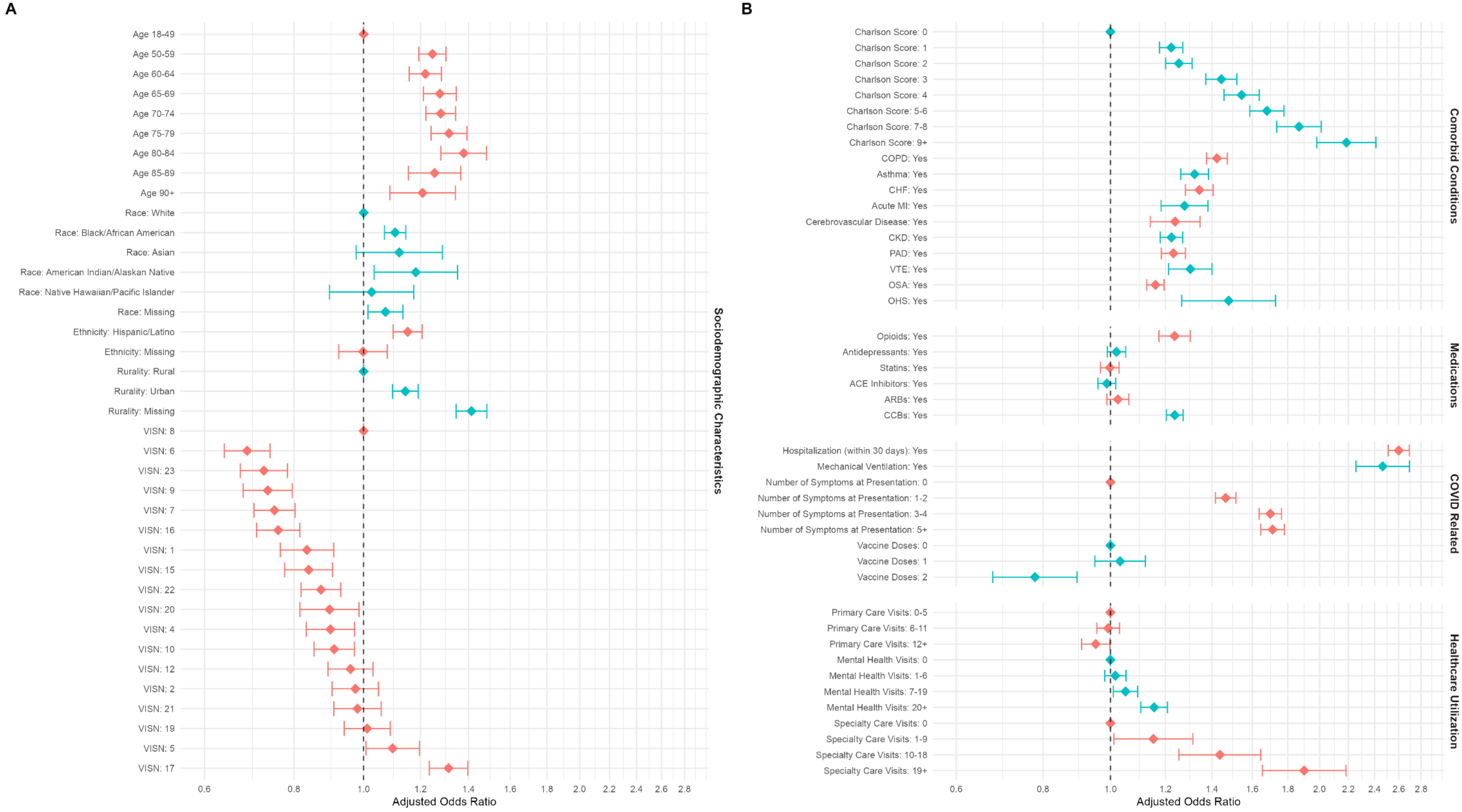

© 2022 Ioannou GN et al. *JAMA Network Open.*
